# Predication of the post mining land use based on random forest and DBSCAN

**DOI:** 10.1371/journal.pone.0287079

**Published:** 2024-01-02

**Authors:** Qiang Bo, Pinhan Lv, Ziguan Wang, Qian Wang, Zechuan Li

**Affiliations:** 1 Kunming University of Science and Technology Oxbridge College, Yunnan, China; 2 The 4th Geological Brigade of Henan Nonferrous Metals Geology and Mineral Bureau, Henan, China; 3 Research Academy of science and culture, China university of Geoscience, Beijing, China; 4 Southwest Survey and Planning Institute of National Forestry and Grassland Administration, Yunnan, China; Jinan University, CHINA

## Abstract

Mine reclamation is one of the most important stages of the mining activities in line with the basic principles of sustainable development. In this study, different post-mining land uses are evaluated in the Hongliulin mining area, which is located in Shen mu country of China. 145 soil samples were collected in the May,2021 by using the soil auger, and the sampling depths were 0–20 cm. The sampling points contains 45 to be reclaimed samples and 100 existing classification land use types. 14 environmental factors including soil organic matter (SOM), total nitrogen (TN), available phosphate (AP), available potassium (AK), K, Slope steepness, curvatures, aspect, length, Topographic Wetness Index (TWI), NDVI and elevation were extracted and calculated based on laboratory test and digital elevation map. The random forest classier showed a great prediction capability, with only 1 miss-classified sample in the validation data-set, the accuracy of the classification model was 95%. The content of TN of C1 is 5 times more than C2 and 4 times more than C3. Also, the K value of C1 column is maximum and over 0.4, which means the soil particle is relatively smaller and the soil texture of it is sandy loam. In terms of the 45 to be reclaimed samples, 15 samples were classified into C1, 23 samples were classified into C2, 5 samples were classified into C3, 2 samples were classified into C4. The value of K and content of soil nutrients of the samples classified to be C1 column(C1-C) is maximum. The soybean and murphy were suggested based on the soil nutrients index and with the mining disturbance on cluster 2 of C1, the ground subsidence filling as well as soil nutrients increased strategy should be applied. The result may contribute to the land use planning and idle land utilization strategy.

## 1. Introduction

Mining activities have resulted in numerous adverse effects on both the land and the economy. The inherent conflict between humanity and the natural environment has grown progressively more pronounced. Currently, around 30% of mining regions in China reached a stage of maturity and decline following an extended period of mining activity. Inefficient utilization of land resources can lead to significant wastage, necessitating the implementation of reclamation as a viable strategy for safeguarding these resources [1]. Simultaneously, it can enhance the land use rate within the mining region, so playing a crucial role in the adjustment of the mining area’s economic structure and the transformation of its growth patterns [2]. In spite of the fact that arable land area has been approaching the "alarming line" in recent years, land use categories in mining areas remain extensive [3].

The assessment of land reclamation necessitates the consideration of two key factors: the soil nutriments index, which encompasses soil organic matter, nitrogen, and phosphorus levels, and the topographic index, which encompasses slope steepness, elevation, and curvature [4]. When developing an evaluation model for the mining area, the initial consideration should be the categorization of land use categories for the land that is to be reclaimed. The mining area in China encompasses four primary categories of land use: cultivated land, shrub land, grassland, arbor land, and solar station [5]. Considering the priority was to reclaim for agricultural purposes, it is essential to assess the soil fertility and arability of the specific area [6]. The occurrence of numerous ground fissures and land deformation resulting from mining operations will lead to the reduction of soil thickness, disruption of soil structure, depletion of soil fertility, and other unfavorable conditions for soil quality [7]. After the mining disturbance, there was a significant loss in soil nutrients, ranging from approximately 50% to 70% [8]. Hence, it is imperative to thoroughly assess the influence of soil nutrients in order to accurately determine the appropriateness of reclamation efforts. If the land was unsuitable for restoration to cultivated land due to limits related to its appropriateness, an assessment of its potential for reclamation into forest land shall be conducted based on its specific site circumstances. Meanwhile, the viability of utilizing reclamation as a solar station could be assessed by considering elements such as the undulation and fragmentation of the landscape. The appropriate reclamation strategy based on varying suitability classifications is essential.

Soil organic matter (SOM) and total nitrogen (TN) was perceived as the most essential nutrients in reclamation process. Soil organic matter is the main source of trace elements, such as nitrogen and phosphorus required by plants. According to statistics, more than 75% of the nitrogen in the topsoil (0-20cm) of the study area exists in the form of organic state. The mineralization of soil organic matter further promotes the degradation and transformation of microorganisms. Microorganisms released nutrients throughout the process of degradation and transformation [9, 10]. The structure of soil will be affected by soil organic matter. During the extensive research on sandy soil, it could be found that organic matter can effectively increase the agglomeration effect of soil particles, making it softer, structured and sticky soil, increasing the tillability of the soil. Nitrogen is an important element of chlorophyll in plants. In addition, nitrogen also plays an important role in the formation of proteins and the pairing of nucleic acids, and can help chlorophyll in the conversion of inorganic matter to organic matter and the conversion of light energy and chemical energy [1]. Meanwhile, the available nutrients such as available phosphorus was crucial for plants and vegetation growth. However, post-mining land use studies often address a range of environmental concerns, such as water quality, habitat restoration, and air pollution. These issues might take precedence over soil-related research, leading to a relative lack of attention to soil organic matter and total nitrogen. Thus, it was necessary to evaluate the SOM, TN and other available nutrients in the reclamation process.

Multi-criteria decision-making (MCDM) has been widely utilized to evaluate the suitably of land reclamation, among these studies, PROMETHEE and TOPSIS was proved to be effective strategy for classification of land use types in terms of reclamation [11]. However, the complexity in the classification of land use type is generally non-linear, thus the MCDM may fail in the accurate classification of land use types as MCDM was only susceptible to linear classification. The utilization of machine learning has demonstrated its effectiveness in addressing the classification of non-linearity data [1]. Meanwhile, the MCDM required the weight of the each indicators to build the model, which was often obtained by the expert scores method. Expert scores are inherently subjective, as they are based on the opinions, experiences, and judgments of individual experts. Different experts may have varying perspectives and biases, which can lead to inconsistent or divergent scores [1]. Expert scoring lacks standardization, meaning there may not be a uniform or objective scale for rating different items. This can make it difficult to compare scores across different assessments or between different experts. In group settings where multiple experts contribute to the scoring process, stronger personalities or dominant individuals may disproportionately influence the final scores, potentially overshadowing more nuanced or diverse perspectives [2]. Whereas, the relatively importance determined by the machine learning algorithm was much more objective, Thus, it was crucial to determine the post mining land use types based on machine learning.

The Hongliulin Coalfield is one of the major coal-producing areas in Shenmu County. It is an essential part of China’s coal industry and contributes to the country’s energy supply. Coalfields like Hongliulin play a crucial role in supporting China’s economic development, as coal remains a primary source of energy for various industries in the country. However, about 30km^2^ of golfs have appeared after the longwall mining disturbance since 2013, and the land is idle. In addition, more working faces are being mined one after another, and a large number of abandoned land will appear [8]. Several severe devastations to the land resource was brought to the land due to the mining activities. 1). About 1/4 area has been proved to have a ground fracture/collapse pit or other forms of subside, which cause the transportation such as bridge and country road stalled or nearly paralyzed. 2). Plentiful cropland has been occupied to serve the mining. Once mining is complete, the coal gangue and the pipeline as well as other facility was deserted, which caused idle land and abandoned land. The major aims of this manuscript were: 1). extract environmental factors affecting post-mining land use classification. 2). build the post-mining land use prediction model based on random forest. 3). decide the specific land use types in cropland and shrubland & grassland by using DBSCAN clustering method.

## 2. Study area

**Fig 1:**
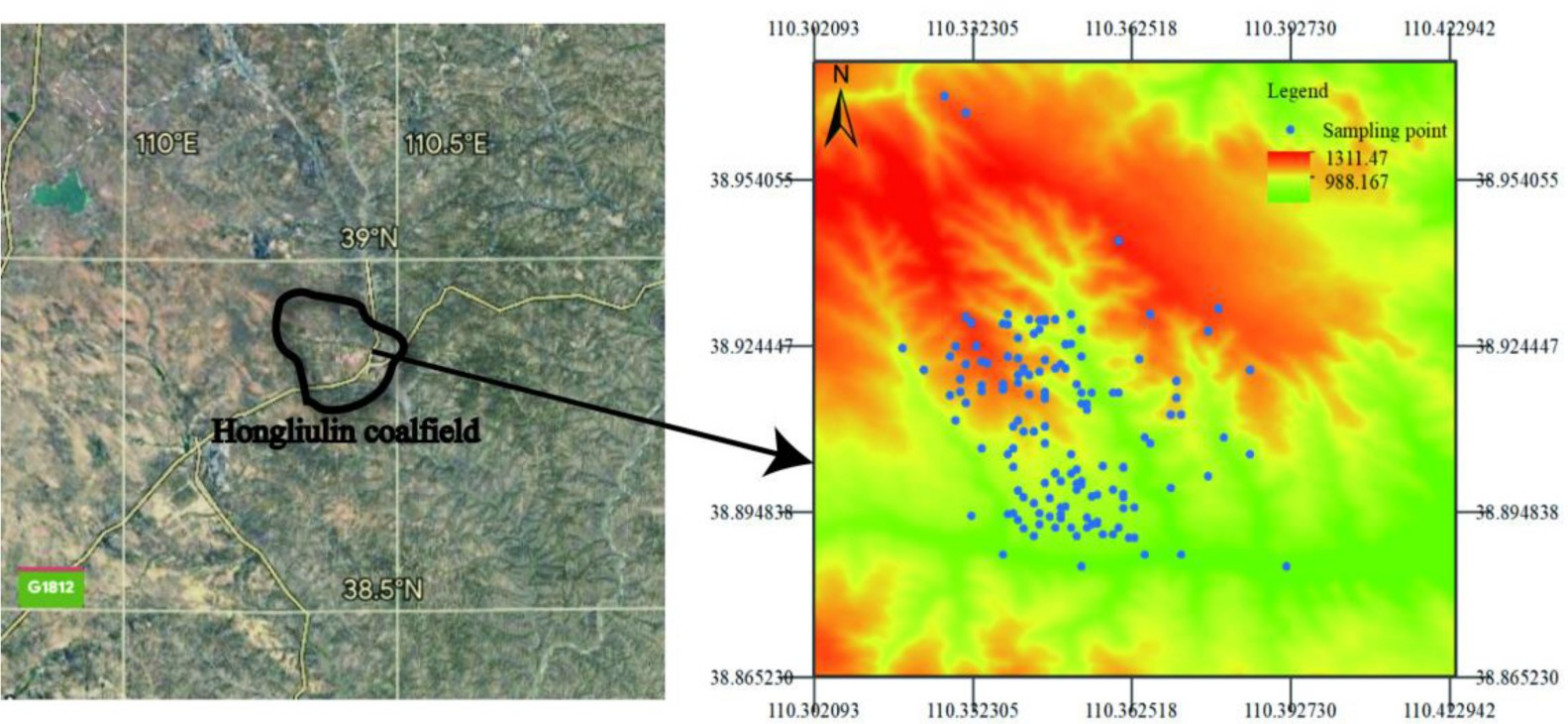
Study area and sampling points.

The area is located in the northern part of the Loess Plateau in northern Shaanxi, on the southern edge of the Mu Us Desert (Fig 1). The western part is a wavy dune land, and the eastern part is a loess geological valley [1]. The study area belongs to the first panel of the Red willow mining area. The terrain is open and the loess gully develops. The terrain is generally high in the northwest, low in the southeast, high in the middle, and low in the north and south. The study area belongs to the mid-temperate semi-arid continental climate, with cold winters and hot summers. The temperature difference between day and night is with huge disparity, about 20°C maximumly. From November to March of the following year, the maximum depth of frozen soil is 146 cm; the maximum snow thickness is 12 cm; the monsoon period from the beginning of January to the beginning of May, mostly northwest wind, the average annual wind speed is 2.5 m/s, and the maximum wind speed is 25 m/ s, the annual average temperature is 8.5°C, the extreme maximum temperature is 38.9°C, the extreme minimum temperature is -28.5°C, the annual average precipitation is 434.10 mm, and the concentration is mostly concentrated in 7, 8 and 9 months; the annual average evaporation is 1907.2 to 2122.7 mm, about 4 to 5 times the amount of precipitation [8].

## 3. Materials and methods

### 3.1. Sampling points and current land use types

The study area has been suffering severe mining disturbance since 2013. 145 soil samples were collected in the May 2021 by using the soil auger, and the sampling depths were 0–20 cm. For each sample, soil from 5 points within a 50 m radius area was taken and put together to make a mixed sample for soil chemical and physical analysis. The sampling points contains 45 to be reclaimed and 100 existing classification land use types. These sampling points were determined by using “random points” section in ArcGIS software and the interval was set as 1 km. The sampling soil type is aeolian sandy soil. All of the sampling points were collected where mining disturbance occurred. In this study, the authority of Houliulin coalfield has approved the field site access.

The samples comprise 4 types of previous land use and now are all idle and destructive due to the mining activity (Table 1). Cropland and shrub land occupy 81% of the total land use types. Among the cropland, 85% of which is cornfield, the other is murphy. The dominated vegetation types are artificial shrubs (shrub sophora, korshinsk peashrub, and sea-buck-thorn), natural grasses (*Artemisia sacrorum*, *Artemisia giraldii*, *Le*-*spedeza daurica*, *Artemisia scoparia*, *Bothriochloa ischaemum*, and *Stipa bungeana*) and shrub (*Periploca sepium*). The features were utilized to establish the classifier comprise terrain factors and soil nutrients.

**Table 1:** Current land use of 100 samples.

Classification	Land use mode	Proportion
C1	Cropland	43%
C2	Shrub and grass land	38%
C3	Arbor land	10%
C4	Solar station	9%

### 3.2. Environmental factors

1) K factor. K factor is one factor embodied in the USLE model and is utilized to evaluate the erodibility of the soil, the formula of the K factor is as below [2]:
$$K=(0.2+\exp(-0.0256SAN(1-\frac{SIL}{100})){\left(\frac{SIL}{CLA+SIL}\right)}^{0.3}$$
$$\left(1-\frac{0.25S}{S+\exp(3.72-2.95S)})(1-\frac{0.7SN1}{SN1+\exp(-5.51+22.9SN1)}\right)$$ (1)
  Where,
  SAN, SIL, CLA, S refer to the percentages of sand, silt, clay and SOM in the soil (%), respectively. SN1 = 1-SAN/100.
  Generally, the K value of sandy loam is higher than the loamy sand due to the particle of it is smaller. The K factor limited an area to be reclaimed for cropland as the large particle will result in the nutrients loss.
2) Topographic Wetness Index. TWI is considered as one quantitative and accurate description of soil moisture content in a small watershed, which proved to be one major limiting factor for agriculture and reclamation in the arid and semiarid area. TWI is calculated as below [1]:
$$TWI=\ln(\underset{CL\to 0}{\lim}\frac{CA}{CL})*\frac{1}{\tan\beta }$$ (2)
  Where,
  CA refers to the flow accumulation and CL refers to the flow direction. β refers to the slope steepness(°). A high value of TWI usually signifies a larger flow confluence area, which lead to a higher soil moisture content and ability to generate runoff on account of more susceptible to achieve saturation.
3) Curvature and NDVI. Generally, profile curvature is parallel to the slope and indicate the direction of the maximum slope. It affects the acceleration and deceleration of the flow through a surface. Plane curvature is a direction perpendicular to the maximum slope. It affects the convergence and dispersion of flows through a surface. A negative value of profile curvature indicates that the surface of the cell is convex upward and the flow rate will decrease. A positive value indicates that the surface opening is concave upwards and the flow rate will increase. A value of zero indicates that the surface is linear. A positive value of plan curvature will separate the runoff will a negative one will accumulate the runoff. the Normalized Difference Vegetation Index (NDVI) can reflect vegetation coverage, which is generally high in the arbor land and cropland in summer [8].

Soil nutrients are taken to evaluate the fundamental property of the post mining land. The analyses items included soil organic matter (SOM), total Nitrogen (TN), available phosphate (AP) and available potassium (AK). SOM was measured with the K_2_Cr_2_O_7_ heating method [1]; TN, with Semi-micro Kjeldahl method [2]; AP, with the alkaline hydrolysis NaHCO3-extraction-Mo-Sb-Vc-colormetry [8]; and AK, with ammonium acetate extracts Flame photometer [1]. The soil texture was measured with the soil sieve. The relative deviation is less than 5%. All of the above components were analyzed at the laboratory of the Chinese Academy of Agricultural Sciences. All of the terrain factors are finished in the Arcgis 10.1 by using a 5-meters digital elevation map.

### 3.3. Random forest classification

Random forest classification is an ensemble learning firstly proposed by Breiman [12] which processes a classifier that utilizes multiple trees to train and predict samples [12]. Simply put, a random forest is made up of multiple CART (Classification and Regression Tree). Classification is a type of supervised learning task where the goal is to predict the category or class label of a given input data point. The input data consists of features or attributes, and each data point is associated with a specific class or category. The task of the classification algorithm is to learn a mapping between the input features and the corresponding class labels based on a training dataset [12]. For each tree, the training set they use is back-sampled from the total training set, which means that some samples in the total training set may appear in the training set of a tree multiple times, or may never appeared in the training set of a tree. When training the nodes of each tree, the features used are randomly extracted from all the features according to a certain proportion. According to Leo Breiman’s suggestion, the total number of features is assumed to be M. This ratio can be sqrt (M), 1/2sqrt(M), 2sqrt(M).

A tree is made up of all of the features (soil nutrients and terrain factors) based on the purity of mathematics, which means target variables must be separated specifically. A tree will try each feature once, and then select the one that can make the best feature of the classification as the parent node. The calculation of the best feature is based on the Gini coefficient, a splitting formula classic binary tree as below:

$$gini_{left/right}=1-\sum pj^{2}=1-\sum {\left(\frac{nj}{s}\right)}^{2}$$ (3)

$$gini_{split}=\frac{S_{left}}{s}*gini_{left}+\frac{S_{right}}{s}*gini_{right}$$ (4)

Where: Pj is the frequency column j appears in the total data set. Nj is the number of column j in the data set. S is the data numbers in the data set. The less Gini coefficient, the higher purity. In each split, the tree will choose the feature which the Gini is minimum as the split node.

A random forest is made up of 100 trees in the present research. A tree is made up of random number of samples and features. By voting result, it is decided by these 100 trees which category the data belongs to (the voting mechanism has one vote veto system, the minority subject to majority, and the weighted majority).

### 3.4. Density-Based Spatial Clustering of Applications with Noise (DBSCAN cluster)

DBSCAN is a density-based clustering algorithm. Such density clustering algorithms generally assume that categories can be determined by the closeness of the sample distribution. Samples of the same category are closely connected, which means there must be samples of the same category not far from any sample in the category. By classifying closely connected samples into one class, a clustering category is obtained. Classify all closely related samples into different categories, and the final results for all cluster categories are obtained. Two fundamental parameters of DBSCAN are EPS(ε) and Main points (MinPts). ε defined the radius around each data point within which the algorithm searches for other points to form a cluster, and MinPts specified the minimum number of data points required within the ε neighborhood of a point to classify it as a core points [1, 8].

A benefit of utilizing DBSCAN is that the cluster is not based on a variety of distance metrics, but on density. Therefore, it can overcome the shortcomings of distance-based algorithms that can only find “circular-like” clusters. Meanwhile, the numbers of the cluster category cannot be set up in advance, which makes it much more objective than other clusters.

## 4. Results

### 4.1. Current land type classification and soil property

As is shown in the Table 2, 82 samples which comprise 4 current land types and are utilized to build the random forest classifier. After the establishment of the model, the gain of each feature is calculated and the other 18 samples will be classified by the model based on the gain of each feature sequentially.

**Table 2:** Mean value of environmental factors of the 100 samples.

Feature	C1(37)	C2 (30)	C3 (9)	C4 (6)
TWI	4.41	5.2	5.786	5.468
NDVI	0.238	0.2	0.287	0.18
Profile curvature	3.94	-13.67	11.43	-1.63
Plan curvature	-4.263	3.784	-18.41	0.32
K	0.412	0.31	0.32	0.32
Slope steepness (°)	5.41	5.09	7.95	4.37
pH	8.67	8.66	8.7	8.7
Slope length (m)	3.001	7.47	25.66	4.2
TN (g/kg)	0.486	0.09	0.12	0.015
AP (mg/kg)	7.87	4.5	3.2	2.47
AK (mg/kg)	97.94	47.34	48.32	40.3
SOM (g/kg)	12.727	9.59	10.32	9.4
Slope aspect	157.5~292.5	112.5~247.5	202.5~292.5	157.5~202.5
Elevation (m)	1173	1169	1147	1174

The 82 samples comprise 32 cropland, 28 Shrub and grass land, 14 arbor land and 8 solar station. As is shown in the table, the soil nutrients comprise TN, AP, AK and SOM are most enriched in C1. The content of TN of C1 is 5 times more than C2 and 4 times more than C3. As is shown in the previous research, TN is the most significant nutrient affecting the cropland [1]. Also, the K value of C1 column is maximum and over 0.4, which means the soil particle is relatively smaller and the soil texture of it is sandy loam. The TWI of C1 is minimum, in consideration of a positive value of profile curvature and a negative value of plan curvature, this is presumably induced by the slope length as the cropland is mostly at the top of the slope the TWI could decrease from the bottom to the top of a slope due to the accumulation of the run off. There are some similarities between C2 and C3 column. TN, AK and SOM in C3 is slightly higher than in C2. In terms of AP, C2 exceeds a little. Also, the K value of these two columns is similar, demonstrating a same type of soil—loamy sand. The results of the curvature indicated that the runoff was accumulated on the slope, which ensures the abundant moisture that arbor requires. While the curvature of C2 could decrease and disperse the run-off, such circumstance of moisture is suitable for the drought enduring shrub and grass.

By contrasting C1, C2 and C3, it can be inferred that the limiting factor of classification for C1 column is TN and K as the murphy and corn require TN as the top priority soil nutrient and loamy soil texture. As for the C2 and C3 column, the major classify factor could be NDVI and curvature. The NDVI of C3 is 0.087% higher than C2 and the curvature of C3 accelerate the runoff well the C2 resists instead. In terms of C4, the construction land, proved to be the minimum content of soil nutrients.

### 4.2. Random forest classification of post mining land

The 82 samples engaged in the construction of the classification model based on the Gini. In each tree, 14 environmental factors and 82 samples are randomly selected and constructed. One of the trees is as below, where 52 samples and 6 features (TN, AK, TWI, Plan curvature, K and slope length) are selected. The Gini is calculated in each split till it reaches to zero, and the split will terminate. The max depth of the tree was set as 4 in case the model is overfitting.

**Fig 2:**
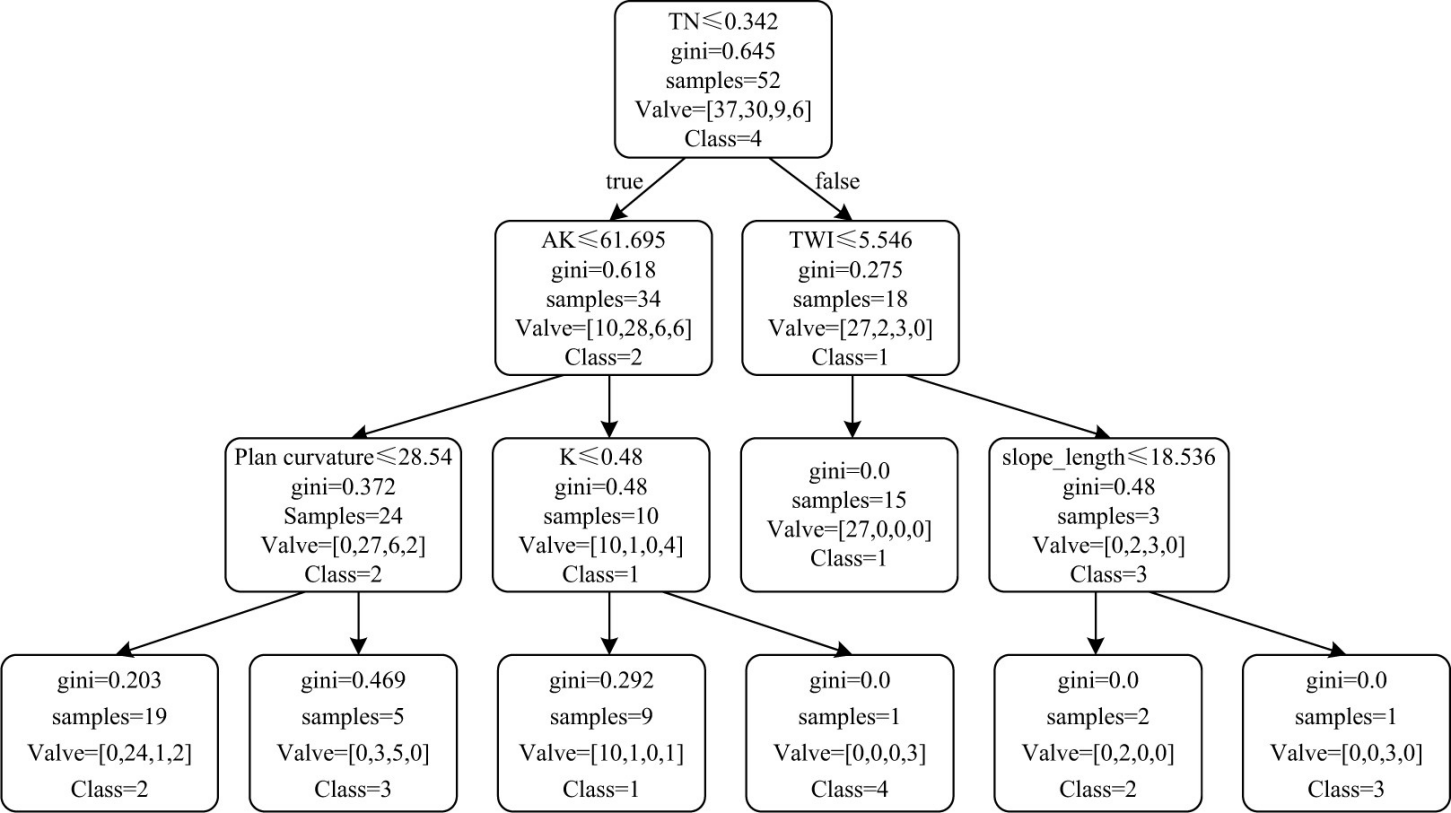
Classification tree build to predict the post-mining land use. (C1: Class = 1, C2: Class = 2, C3: Class = 3, C4: Class = 4).

As is shown in the Fig 2, The first parent note which classified C1 and C2 is TN. The left branch continues to classify the C1 and C2 with the criteria of AK while the right branch classifies C1 and C3 with the criteria of TWI. When the depth goes to 3, the left branch of true path classifies C2 and C3 with the criteria of plan curvature and the right branch classifies C1 and C4 with the criteria of K. The left branch of the false path terminates and the right branch classifies C2 and C3 with the criteria of slope length.

As the tree demonstrates, TN and AK are two major criteria to classify C1 and C2, which indicates that theses two soil nutrients are with highly distinction in term of cropland and shrub land among the selected 6 features. While, the TWI is major criteria to classify C1 and C3, indicating that a catchment area could conspicuously differentiate cropland and arbor land as the GINI decreased to zero. In the third depth, the plan curvature which distinguish the shrub land and arbor land is not efficient as the second depth, as in each branch the C2 and C3 consists in. The K value is selected to classify shrub land and solar station, while the slope length is selected to classify shrub land and arbor land, which is generally accord with the previous data analysis. From the classifier tree, it can be inferred that C3 is literally distributed in the middle to the bottom of the slope and with high value of TWI. C2 is basically distributed in the upper and middle of the slope.

### 4.3. Classification for the test and train index

18 samples are utilized to examine the precision of the classification model as validation data-set. As is shown in the Table 3 below, the accuracy of the prediction model is about 95%, which is perceived as an accuracy model.

**Table 3:** Validation of the post-mining land use classification.

Samples Number	Reality result	RF prediction
11	C4	C4
19	C2	C2
25	C2	C2
32	C2	C2
42	C2	C2
45	C1	C1
47	C3	C2
49	C4	C4
***55***	***C2***	***C1***
66	C4	C4
67	C2	C2
73	C1	C1
75	C1	C1
83	C2	C2
84	C2	C2
85	C1	C1
91	C1	C1
98	C1	C1

Sample 55 is the only sample that was misclassified by the random forest. It was supposed to be in class C2, but the random forest categorized it as class C1. However, the soil property of No.55 is ambiguous for the classifier as below: TWI = 5.711, NDVI = 0.286, profile curvature = 7.97, plan curvature = -11.8391, K = 0.42, Slope steepness = 4.6, pH = 8.6, slope length = 18.45, height = 1213, TN = 0.02, AP = 2.75, AK = 42.895, SOM = 12.91, slope aspect = 296.551. This sample is normally perceived as the noise point as the soil nutrients are basically above the average except the content of TN. The K value is literally attributed to the C1 while the NDVI attributed to C2. The Gini coefficient which a purity criterion selected is incapable to classify this sample.

The 45 samples are totally idle post mining land and were predicted based on the classifier. As is shown in the Table 4, 15 samples were classified into C1, 23 samples were classified into C2, 5 samples were classified into C3, 2 samples were classified into C4. The value of K and content of soil nutrients of the samples classified to be C1 column(C1-C) is maximum. Simultaneously, the slope steepness (°) is below 5° and is perceived as gentle surface, which is literally appropriate for reclaiming to cropland. C2-C, a relatively high content of soil available nutrients and the minimum value of K, is suitable for the shrub. Considering the low value of NDVI, the samples to be reclaimed to shrub and grass land could contribute to the ecological restoration by decreasing the soil erosion at the upper slope. Five samples are classified to the C3-C and the slope length of which is maximum while the content of TN and SOM is relatively high. which is accord with the circumstance that the site afforestation requires, the sunny slope could also ensure the sunshine and temperature condition for arbor. The two samples which possess the minimum content of soil nutrients and slope steepness are classified to C4-C. A relatively flat and smooth surface and a sunny slope is suitable for constructing the solar station.

**Table 4:** Post-mining land use prediction and the responding environmental factors.

	C1-C(15)	C2-C(23)	C3-C(5)	C4-C(2)
TWI	4.2	5.1	5.93	5.4
K	0.41	0.18	0.24	0.34
TN (g/kg)	0.28	0.038	0.056	0.027
AP (mg/kg)	5.55	3.67	2.2	1.5
AK (mg/kg)	97.75	42.477	39.499	29.648
SOM(g/kg)	13.988	8.79	9.93	8.31
Slope steepness (°)	4.32	5.57	4.14	2.18
Slope length (m)	3.31	7.62	19.36	1.25
NDVI	0.18	0.13	0.18	0.11
Profile curvature	3.57	-5.05	0.2126	0.16
Plane curvature	2.006	6.2	-0.012	-0.01

By the application of the Random Forest, 45 samples were classified as 15 C1, 23 C2, 5 C3 and 2 C4. The proportion of the classification does not match to the initial proportion (37 C1, 30 C2, 9 C3 and 6 C4), indicating that the original proportion is not the fundamental of the classification. Unlikely to the neural network and SVM, the Gini of the random forest increased the accuracy and objectivity.

### 4.4. DBSCAN cluster utilization to the C1 and C2

In 158 samples, the random forest classified 58 of them into class C1 and 61 of them into class C2. A DBSCAN cluster is utilized to analyze the coupling effect between soil property and the terrain distribution so that the specific plants and crops to be reclaimed or the quality and suitability of the same cluster could be determined. For C1, the local crops include maize and potatoes. The essential nutrients required for growing these crops are SOM, TN, and AP. Therefore, choosing these three indicators for clustering can grade the nutrient quality of the soil, thus determining whether further soil remediation and nutrient cultivation are necessary. When EPS is set as 3, C1 is separated into two clusters, Cluster 1 comprises 24 samples and the average content of TN, AP and SOM is 0.566 g/kg, 11.93 mg/kg and 19.975 g/kg respectively, while Cluster 2 comprises 34 samples and the average content of TN, AP and SOM is 0.304 g/kg, 3.12 mg/kg and 8.36 g/kg respectively.

**Fig 3:**
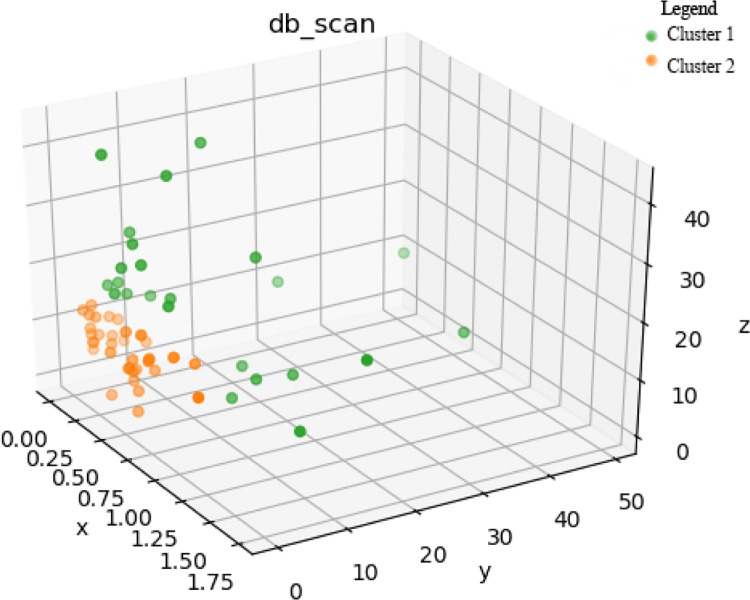
DBSCAN clustering based on SOM, TN and AP for C1.

As is shown in the Fig 3, x, y and z refers to the TN, AP and SOM. The green points (Cluster 1) encircle the orange points (Cluster 2), The result of the cluster could also be proved by the content of the AK in which the content of C1 is average 112 mg/kg while C2 is 81 mg/kg. It can be inferred that the Cluster 1 and Cluster 2 of C1 are two different scale of soil fertility.

**Fig 4:**
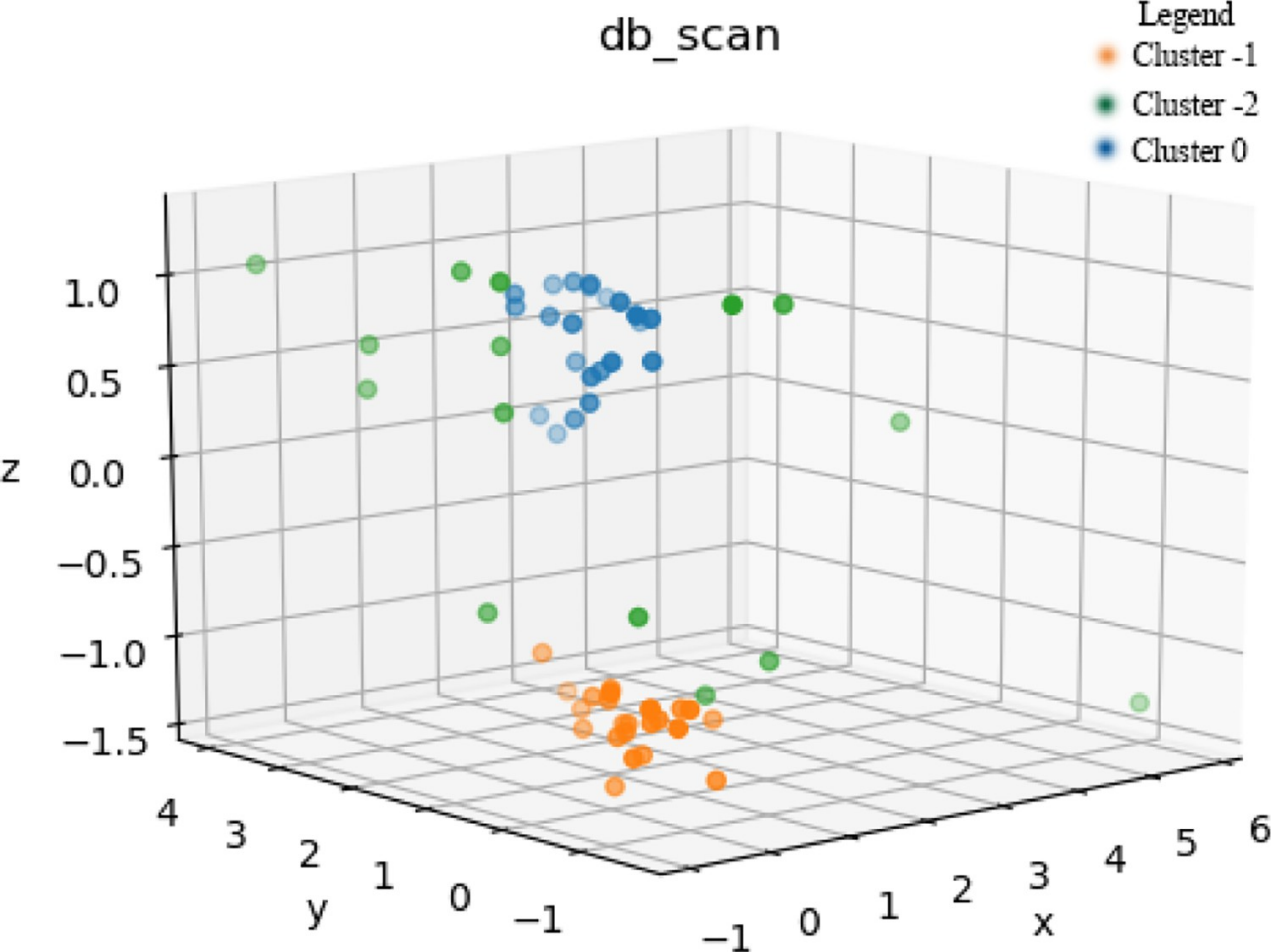
DBSCAN clustering based on AP and AK for C2.

As is shown in the Fig 4, x, y and z refers to AP, AK and K. When EPS is set as 2, C2 is assembled by 3 parts. Cluster -1 comprises 24 samples and the average value of AP, AK and K is 3.4 mg/kg, 34.95 mg/kg and 0.21. Cluster 0 comprise 22 samples and the average value of AP, AK and K is 3.09 mg/kg, 46.07 mg/kg and 0.45. The criteria of orange points (Cluster -1) and blue points (Cluster 0) is K. Cluster -2 comprises 15 samples and average value of AP, AK and K is 6.6 mg/kg, 58.53 mg/kg and 0.37 respectively, in which the content of AP and AK is maximum and SOM is minimum.

## 5. Discussion

### 5.1. The accuracy of random forest algorithm and its superiority

As is shown in Table 3, only one sample was mis-classified, demonstrating a high accuracy of the clarification algorithm. Compared to the traditional MCDM, the classification algorithm could learn the previous current land use types and its environmental factors similarity. Furthermore, the validation data set could provide the evidence whether the land use types were properly classified, which was the superiority that MCDM could not process [13–15]. Random Forest is an ensemble learning method that combines multiple decision trees to form a strong and diverse model. Each decision tree in the forest is trained on a random subset of the data and a random subset of features. This randomness and diversity help reduce overfitting and improve the generalization performance of the post-mining land use types [12, 16]. Random Forest employs a technique called bagging, where it randomly samples the training data with replacement to create different subsets of data for each tree. This sampling process introduces diversity, reducing the risk of overfitting and increasing the overall accuracy of the ensemble in terms of the post-mining land use types [17]. In addition to data sampling, Random Forest introduces feature randomness. At each node of the decision tree, a random subset of features is chosen rather than all of the features. In comparison to the MCDM, each environmental factors could be chosen as a node; however, in the MCDM, if the weights were low scored by the experts, it rarely contributed to the final rankings. As a result, the random forest algorithm was much more objective than the MCDM, which contributed to the high accuracy of the classification results. This feature subsampling further enhances the diversity of the trees and helps capture different patterns in the data [13, 18]. During prediction, each decision tree in the random forest independently makes its prediction. The final classification is determined by a majority vote or averaging of the individual tree predictions. This voting mechanism tends to reduce the impact of individual noisy or misclassified data points, leading to more accurate overall predictions [19].

### 5.2. The specific land planning on C1 and C2

Generally, Cluster 1 of C1 is suitable for cultivating the corn and murphy or soybean while slightly lack of TN and urea could be applied under certain circumstances. In terms of Cluster 2, the soil nutrients are comparatively barren in which manure and aquasorb is required. Ammonium phosphate and farmyard manure could be utilized to ameliorate the soil fertility.

**Fig 5:**
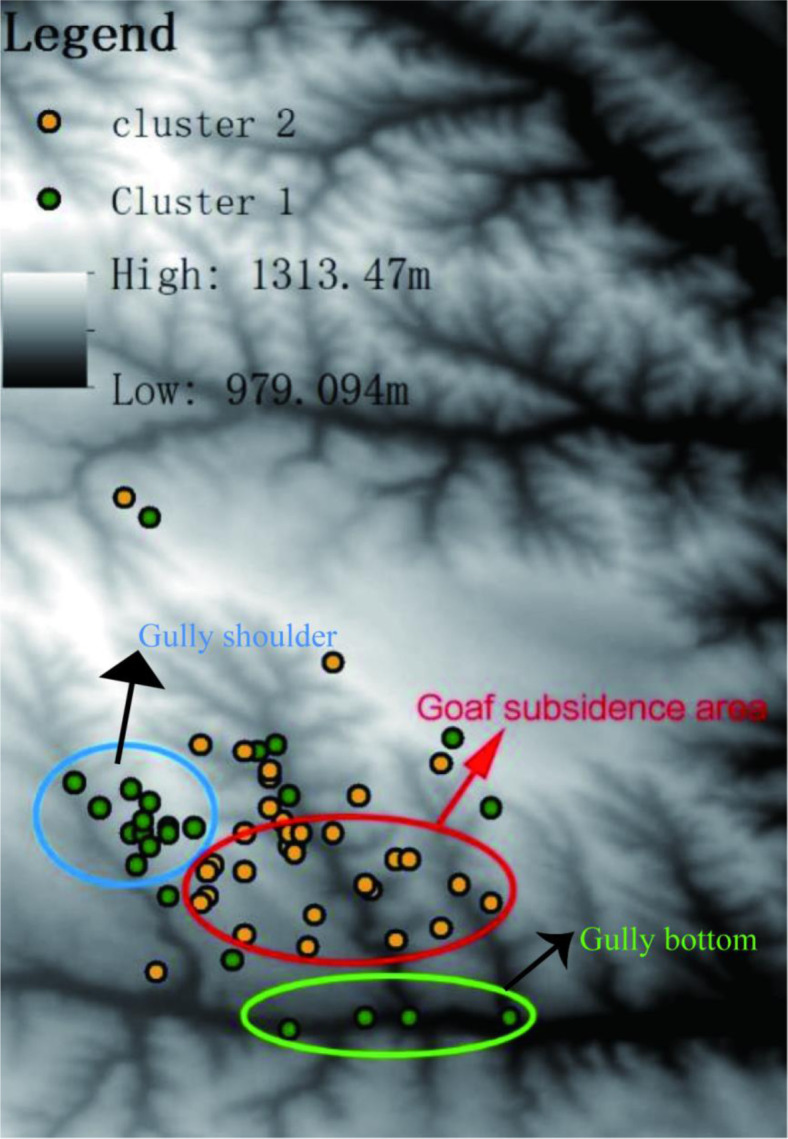
Spatial distribution of C1.

As is shown in the Fig 5, the orange points (cluster 2) are basically distributed in the mined-out subsidence area and green points (cluster 1) are basically distributed in the non-collapsed area and valley, which evidently demonstrates that ground fissures and collapse pit has a significantly negative effect on the soil nutrients. In this situation, ultra-high-water material filling could be applied: Firstly, some coal gangue and loess with smaller particle size are used for deep filling. When the bottom crack is closed, the ultra-high-water material (bauxite and gypsum) is used for filling, of which 90% the content is water. After fully mixing the ground crack, the material quickly condenses within a few hours. At this time, the crack was filled with the soil nearby and the thickness was 50 cm. The planting of maize and potatoes could be implemented in Cluster 2 after the cracks are filled.

Land subsidence and the ground fissures on the goaf area significantly affect the soil nutrients and thus post-mining land use types. Land subsidence often results in the compaction and consolidation of soil layers, leading to decreased pore spaces and a reduction in soil organic matter content. As subsidence compresses the soil, it can limit the accumulation of organic debris, such as plant litter and root materials, which are essential sources of organic matter and nutrients for soil fertility [1]. Subsidence can alter soil structure and porosity, affecting water retention and drainage. Compacted soils may have reduced water-holding capacity, which can impact microbial activity and nutrient availability to plants. Subsidence can influence soil chemistry, including pH levels and nutrient availability. In some cases, subsidence can result in soil alkalization or acidification, affecting the solubility and availability of certain nutrients [3]. Subsidence can disrupt soil microbial communities and microbial activity. Microorganisms play a crucial role in nutrient cycling processes, and disturbances caused by subsidence can impact their ability to decompose organic matter and release nutrients. Land subsidence can affect plant root systems and nutrient uptake [2]. Changes in soil physical properties and nutrient availability may impact the ability of plants to access and utilize essential nutrients. Subsidence may alter the topography and surface water flow, potentially leading to increased erosion. Erosion can remove nutrient-rich topsoil, reducing nutrient content and fertility in the affected areas [1]. Table 4 illustrates that the land to be recovered into C1 had the greatest K value, indicating that the area was susceptible to soil erosion. Thus, corresponding soil and water conservation project should be implemented in this area. The surface of the soil should be covered with crops or others to protect the topsoil from erosion.

**Fig 6:**
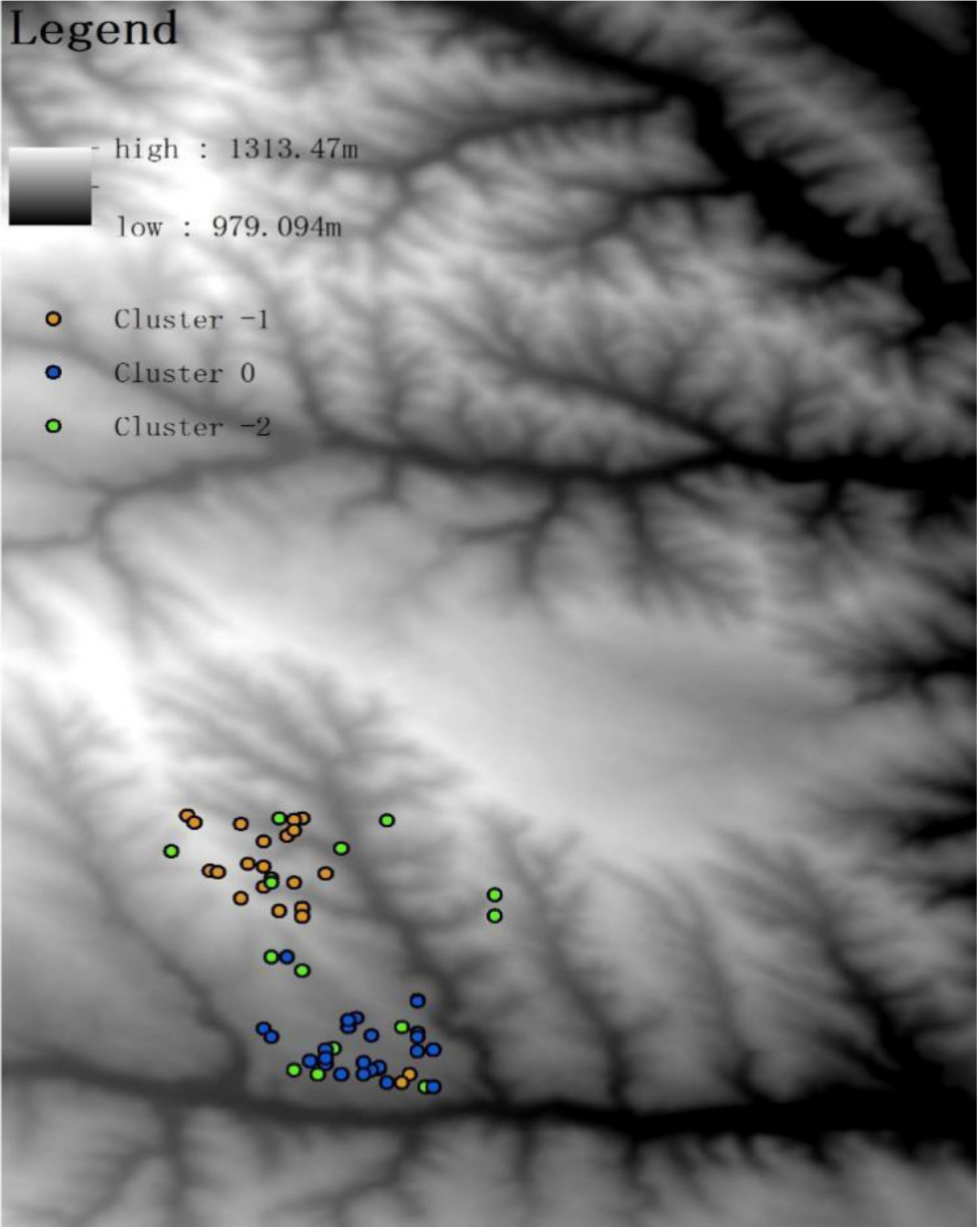
Spatial distribution of C2.

As is shown in the Fig 6, Cluster -1 is basically distributed along the gully while Cluster 0 is distributed in the area with scarcely any gully. Such terrain distribution is presumably a reflection of the soil erosion consequence. In the gully area, large runoff induces the entrainment of small particles of the soil, and the large particles of soil left at the Cluster -1 result in a lower K value. Simultaneously, the runoff of Cluster 0 with barely gully is relatively small, so the small particles result in a higher K value. The AP values of Cluster -1 and 0 are similar. This is induced by the relatively low availability of phosphorus and both two Clusters are therefore less affected by water erosion. Based on the terrain distribution, it can be inferred that Cluster 0 is more suitable for planting grass plants, while Cluster 1 is more suitable for shrubs. According to the previous research, AP and AK are the main nutrients C2 requires and slope aspect, slope steepness and K are three major factors limiting the site condition. As is analyzed, the slope steepness is generally below 10° and none of the slope is shady-slope or semi-shady slope. Therefore, the cluster index of C2 is selected as AP, AK and K. Data standardization is required as the unit of these three indexes are not uniform.

The distribution of Cluster 2 is generally scattered (Fig 6). The abundant soil available nutrients are ideal for leguminous plants. As is demonstrated in the previous research, the leguminous such as alfalfa and sweet clover requires abundant AP and AK to germinate. With its grow, it can gradually increase the content of SOM and be utilized into another shrub or arbor land.

## 6. Conclusion

In this study, 145 soil samples contains 45 to be reclaimed and 100 existing classification land use types were collected in order to build the post-mining land use classification model. The content of TN of C1 is 5 times more than C2 and 4 times more than C3. The accuracy of the prediction model is about 95%, which is perceived as an available model. The bagging strategy, and the voting mechanism contributed to the superiority of the random forest model, making it much more objective than the MCDM. In addition, total of 58 samples of C1 were clustered based on SOM, TN and AP. Soil nutrients in cluster 2 of C1 was significantly lower than cluster 1, demonstrating the effect of land subsidence and ground fissures on nutrients loss. Ammonium phosphate and farmyard manure could be utilized to ameliorate the soil fertility. The land to be recovered into C1 had the greatest K value, indicating that the area was susceptible to soil erosion. Thus, the corresponding soil and water conservation strategy should be applied to this area.

## Supporting information

S1 Data Minimal dataset. (DOCX)
