# Supplementary material for: Predication of the post mining land use based on random forest and DBSCAN
Source: PLoS One. 2024 Jan 2;19(1):e0287079. doi: 10.1371/journal.pone.0287079 (PMC10760759; doi:10.1371/journal.pone.0287079)
Supplement: S1 Data — (DOCX) [file pone.0287079.s001.docx]

Data Availability statement

1. Sharing codes：

Random forest：

# Import necessary libraries

import numpy as np

from sklearn.datasets import load_iris

from sklearn.model_selection import train_test_split

from sklearn.ensemble import RandomForestClassifier

from sklearn.metrics import accuracy_score, classification_report

# Load dataset

data = load_iris()

X = data.data

y = data.target

# Split data into training and testing sets

X_train, X_test, y_train, y_test = train_test_split(X, y, test_size=random(0.15,0.3), random_state= 1 )

# Create Random Forest classifier

clf = RandomForestClassifier(n_estimators=100, random_state=1，max_ depth=4)

# Train the classifier

clf.fit(X_train, y_train)

# Predictions

y_pred = clf.predict(X_test)

# Evaluate classifier

print("Accuracy:", accuracy_score(y_test, y_pred))

print("\nClassification Report:\n", classification_report(y_test, y_pred))

DBSCAN：

import numpy as np

import matplotlib.pyplot as plt

from sklearn.datasets import load_iris

from sklearn.preprocessing import StandardScaler

from sklearn.cluster import DBSCAN

# Load data

data = load_iris()

X = data.data

# It's a good practice to standardize the data for DBSCAN

X = StandardScaler().fit_transform(X)

# Apply DBSCAN

db = DBSCAN(eps=3, min_samples=5)

labels = db.fit_predict(X)

# Number of clusters in labels, ignoring noise if present.

n_clusters_ = len(set(labels)) - (1 if -1 in labels else 0)

n_noise_ = list(labels).count(-1)

print(f'Estimated number of clusters: {n_clusters_}')

print(f'Estimated number of noise points: {n_noise_}')

# Plot the result

plt.scatter(X[:, 0], X[:, 1], c=labels, cmap='viridis')

plt.title("DBSCAN Clustering")

plt.show()

1. Minimal dataset (45 samples to be reclaimed)

| ID | pH | SOM | TN | AP | AK | soil particles(mm) | | | | | | | |
| --- | --- | --- | --- | --- | --- | --- | --- | --- | --- | --- | --- | --- | --- |
|  |  | g/kg | g/kg | mg/kg | mg/kg | <0.002 | <0.005 | 0.05～0.005 | 0.1～0.05 | 0.25～0.1 | 0.5～0.25 | 1.0～0.5 | 2.0～1.0 |
| 1 | 8.79 | 11.10 | 0.46 | 1.84 | 69 | 0.12 | 1.01 | 13.53 | 30.86 | 42.94 | 9.79 | 1.88 | 0 |
| 2 | 8.41 | 6.73 | 0.30 | 1.43 | 42 | 0.00 | 0.00 | 1.63 | 13.48 | 58.41 | 24.48 | 2.00 | 0 |
| 3 | 8.35 | 8.59 | 0.47 | 4.20 | 78 | 1.70 | 3.17 | 24.39 | 31.33 | 31.25 | 8.15 | 1.72 | 0 |
| 4 | 8.81 | 13.78 | 0.49 | 2.43 | 56 | 0.67 | 1.57 | 12.65 | 13.44 | 22.80 | 34.35 | 15.19 | 0 |
| 5 | 8.37 | 19.14 | 0.71 | 7.18 | 77 | 0.03 | 0.80 | 14.13 | 30.37 | 40.12 | 12.56 | 2.03 | 0 |
| 6 | 8.85 | 24.69 | 1.08 | 1.75 | 111 | 0.03 | 0.96 | 14.65 | 22.16 | 39.22 | 19.36 | 3.66 | 0 |
| 7 | 8.70 | 15.25 | 0.18 | 8.29 | 78 | 1.71 | 3.24 | 21.33 | 27.09 | 32.09 | 13.09 | 3.17 | 0 |
| 8 | 8.78 | 10.41 | 0.43 | 2.47 | 62 | 0.03 | 0.92 | 14.20 | 23.02 | 40.12 | 16.36 | 5.40 | 0 |
| 9 | 8.58 | 17.64 | 0.79 | 2.89 | 95 | 1.58 | 2.87 | 29.32 | 31.24 | 24.97 | 8.95 | 2.65 | 0 |
| 10 | 8.78 | 10.33 | 0.51 | 7.01 | 103 | 0.00 | 0.67 | 12.91 | 24.47 | 39.73 | 18.44 | 3.78 | 0 |
| 11 | 8.71 | 3.55 | 0.36 | 3.59 | 52 | 1.09 | 1.98 | 20.86 | 32.66 | 29.92 | 11.71 | 2.88 | 0 |
| 12 | 7.69 | 7.17 | 0.14 | 3.82 | 194 | 2.17 | 4.30 | 31.21 | 32.63 | 24.68 | 5.43 | 1.75 | 0 |
| 13 | 8.82 | 1.43 | 0.33 | 0.72 | 62 | 1.39 | 2.68 | 18.81 | 24.33 | 34.43 | 15.91 | 3.83 | 0 |
| 14 | 8.74 | 4.08 | 0.45 | 0.43 | 56 | 1.89 | 3.38 | 38.34 | 31.71 | 17.09 | 6.96 | 2.52 | 0 |
| 15 | 8.74 | 4.76 | 0.32 | 4.61 | 69 | 2.04 | 3.96 | 32.98 | 31.00 | 24.76 | 5.78 | 1.53 | 0 |
| 16 | 8.72 | 1.72 | 0.30 | 1.50 | 138 | 2.56 | 4.81 | 36.59 | 30.35 | 21.14 | 5.50 | 1.61 | 0 |
| 17 | 8.82 | 5.74 | 0.26 | 0.71 | 84 | 3.32 | 6.09 | 48.95 | 33.21 | 10.34 | 0.79 | 0.62 | 0 |
| 18 | 8.87 | 7.78 | 0.46 | 1.39 | 66 | 2.13 | 3.89 | 30.15 | 31.81 | 24.18 | 7.30 | 2.67 | 0 |
| 19 | 8.93 | 20.65 | 0.42 | 0.41 | 79 | 1.05 | 1.92 | 18.66 | 38.51 | 34.39 | 4.60 | 1.93 | 0 |
| 20 | 8.73 | 9.05 | 0.33 | 0.79 | 116 | 2.29 | 4.27 | 33.06 | 39.18 | 19.52 | 2.36 | 1.61 | 0 |
| 21 | 9.02 | 0.80 | 0.52 | 1.64 | 75 | 1.51 | 2.43 | 29.73 | 44.45 | 21.34 | 1.23 | 0.82 | 0 |
| 22 | 9.06 | 24.14 | 0.87 | 1.16 | 102 | 0.00 | 0.57 | 15.25 | 28.18 | 30.53 | 17.82 | 7.65 | 0 |
| 23 | 9.11 | 5.93 | 0.44 | 5.50 | 90 | 0.00 | 0.00 | 5.54 | 39.63 | 47.97 | 5.32 | 1.54 | 0 |
| 24 | 8.69 | 7.77 | 0.50 | 1.45 | 105 | 1.59 | 3.04 | 26.45 | 29.25 | 28.96 | 9.71 | 2.58 | 0 |
| 25 | 8.93 | 10.25 | 0.48 | 0.60 | 78 | 1.07 | 2.03 | 17.96 | 29.54 | 36.69 | 10.67 | 3.12 | 0 |
| 26 | 8.87 | 12.74 | 0.64 | 1.56 | 125 | 1.27 | 2.20 | 22.69 | 38.69 | 33.06 | 2.75 | 0.62 | 0 |
| 27 | 8.72 | 9.98 | 0.58 | 1.57 | 102 | 2.42 | 4.40 | 38.52 | 32.84 | 20.66 | 2.87 | 0.71 | 0 |
| 28 | 8.69 | 10.74 | 0.61 | 1.46 | 150 | 1.22 | 2.26 | 19.60 | 28.77 | 33.84 | 12.57 | 2.97 | 0 |
| 29 | 8.85 | 11.11 | 0.32 | 0.84 | 74 | 1.28 | 2.24 | 25.61 | 33.53 | 26.18 | 10.01 | 2.42 | 0 |
| 30 | 8.75 | 3.52 | 0.52 | 1.25 | 146 | 1.98 | 3.84 | 30.43 | 32.73 | 23.98 | 6.53 | 2.49 | 0 |
| 31 | 8.75 | 5.50 | 0.48 | 2.32 | 75 | 1.94 | 3.44 | 28.09 | 34.67 | 27.63 | 4.78 | 1.39 | 0 |
| 32 | 8.80 | 6.70 | 0.43 | 8.19 | 76 | 0.99 | 1.94 | 15.34 | 26.77 | 36.84 | 14.13 | 4.98 | 0 |
| 33 | 8.92 | 6.55 | 0.63 | 1.84 | 107 | 1.38 | 2.45 | 30.51 | 35.38 | 26.20 | 4.17 | 1.28 | 0 |
| 34 | 8.75 | 25.79 | 1.74 | 14.84 | 206 | 0.47 | 0.79 | 18.46 | 41.50 | 28.85 | 7.58 | 2.83 | 0 |
| 35 | 8.89 | 11.46 | 0.40 | 2.07 | 65 | 0.94 | 1.90 | 17.03 | 33.80 | 43.06 | 3.50 | 0.72 | 0 |
| 36 | 8.84 | 15.20 | 0.73 | 15.96 | 83 | 1.04 | 1.95 | 19.75 | 29.76 | 34.55 | 11.10 | 2.90 | 0 |
| 37 | 8.67 | 27.71 | 1.12 | 5.20 | 111 | 1.47 | 2.90 | 27.33 | 34.18 | 27.70 | 6.64 | 1.25 | 0 |
| 38 | 8.66 | 42.83 | 1.70 | 6.05 | 123 | 0.67 | 1.28 | 21.09 | 35.35 | 28.90 | 10.64 | 2.74 | 0 |
| 39 | 8.64 | 26.18 | 1.02 | 16.42 | 138 | 1.49 | 2.85 | 28.30 | 35.14 | 25.00 | 6.89 | 1.81 | 0 |
| 40 | 8.55 | 29.40 | 1.02 | 11.07 | 230 | 0.12 | 1.02 | 17.40 | 28.95 | 34.92 | 14.26 | 3.46 | 0 |
| 41 | 8.44 | 38.79 | 1.07 | 41.14 | 81 | 2.16 | 4.42 | 31.96 | 32.08 | 26.10 | 4.64 | 0.80 | 0 |
| 42 | 8.75 | 11.65 | 0.47 | 2.56 | 29 | 0.61 | 1.49 | 13.57 | 13.59 | 19.00 | 33.49 | 18.86 | 0 |
| 43 | 8.63 | 22.82 | 0.86 | 10.07 | 147 | 1.86 | 3.45 | 29.15 | 31.78 | 26.03 | 7.60 | 1.99 | 0 |
| 44 | 8.53 | 13.25 | 0.64 | 15.18 | 116 | 2.66 | 5.18 | 30.42 | 32.17 | 24.41 | 5.93 | 1.89 | 0 |
| 45 | 8.82 | 26.66 | 0.39 | 2.56 | 57 | 0.00 | 0.46 | 7.10 | 19.79 | 37.38 | 24.06 | 11.21 | 0 |
